# Predicting the response to sorafenib in hepatocellular carcinoma: where is the evidence for phosphorylated extracellular signaling-regulated kinase (pERK)?

**DOI:** 10.1186/1741-7015-7-42

**Published:** 2009-08-24

**Authors:** Andrew X Zhu

**Affiliations:** 1 Massachusetts General Hospital Cancer Center, Harvard Medical School, Boston, MA, USA

## Abstract

The approval of sorafenib and active development of many other molecularly targeted agents in hepatocellular carcinoma (HCC) have presented a challenge to understand the mechanism of action of sorafenib and identify predictive biomarkers to select patients more likely to benefit from sorafenib. The preclinical study by Zhang and celleagues published this month in *BMC Medicine *provides preliminary evidence that baseline phosphorylated extracellular signaling-regulated kinase (pERK) may be a relevant marker to reflect the level of constitutive activation of the RAF/mitogen-activated protein kinase kinase (MEK)/ERK signaling pathway and has the potential value in predicting response to sorafenib. The clinical data from the initial single arm phase II study and preliminary report from the randomized phase III study also suggest the correlation of baseline archived tumor pERK levels and time to tumor progression in HCC patients. Whether baseline pERK will prove to be a useful predictive biomarker of response and clinical benefits for sorafenib in HCC will need to be validated in future large prospective studies.

## Commentary

In 2007, Llovet and colleagues first reported the results of the international, phase III, placebo-controlled Sorafenib HCC Assessment Randomized Protocol (SHARP) trial, which demonstrated a longer overall survival (OS) time and time to tumor progression (TTP) compared to placebo in patients with advanced hepatocellular carcinoma (HCC) [1]. As shown in Table 1, the median OS time was 10.7 months in the sorafenib group and 7.9 months in the placebo group (hazard ratio for the sorafenib group, 0.69; *P *< 0.001). The median TTP was 5.5 months in the sorafenib group and 2.8 months in the placebo group (*P *< 0.001). In a subsequent randomized phase III study conducted in Asia, sorafenib also demonstrated a longer OS and TTP in patients with advanced HCC [2]. These studies have led to the timely approval of sorafenib in HCC in many countries worldwide. The development of sorafenib in HCC has several important implications. First, it validates the use of molecularly targeted agents in HCC. Second, it sets a new standard for ongoing and future clinical trials in advanced HCC. Now, the challenge is to understand the mechanism of action of this targeted agent and identify predictive biomarkers to select patients more likely to benefit from sorafenib. In an article published this month in *BMC Medicine*, Zhang and colleagues report their study assessing the potential role of phosphorylated extracellular signaling-regulated kinase (pERK) as a predictive marker in a preclinical HCC model [3].

**Table 1:** Sorafenib HCC Assessment Randomized Protocol (SHARP) [1] and Asia-Pacific [2] study

	SHARP [1]		Asia-Pacific [2]	
Endpoint	Sorafenib vs placebo		Sorafenib vs placebo	

	Hazard ratio (95% CI)	*P *value	Hazard ratio (95% CI)	*P *value

OS	10.7 vs 7.9 months, 0.69 (0.55 to 0.87)	< 0.001	6.5 vs 4.2 months, 0.68 (0.50 to 0.93)	0.014
TTSP	1.08 (0.88 to 1.31)	0.768	0.90 (0.67 to 1.22)	0.50
TTP	5.5 vs 2.8 months, 0.58 (0.45 to 0.74)	< 0.001	2.8 vs 1.4 months, 0.57 (0.42 to 0.79)	< 0.001
RR	2% vs 1%		3.3% vs 1.3%	

OS = overall survival; RR = response rate; TTP = time to tumor progression; TTSP = time to symptomatic progression.

Sorafenib is an oral multikinase inhibitor that targets tumor cell proliferation and tumor angiogenesis by inhibiting the serine/threonine kinases Raf-1 and B-Raf through the canonical Raf/mitogen-activated protein kinase/extracellular signal-regulated kinase (RAF/mitogen-activated protein kinase kinase (MEK)/ERK) signaling pathway and the receptor tyrosine kinases (RTK) of vascular endothelial growth factor receptor (VEGFR)-1, VEGFR-2, VEGFR-3 and platelet-derived growth factor receptor (PDGFR)α and β and stem cell factor receptor (KIT) [4,5]. Human HCC tumors have high expression and enhanced activity of mitogen-activated protein kinase (MAPK) compared to the adjacent non-neoplastic liver [6]. Furthermore, treatment of HCC cells with a MEK inhibitor reduces cell proliferation and induces apoptosis [7], whereas overexpression of activated MEK1 in HepG2 cells enhances tumor growth *in vivo *[8]. Unlike malignant melanoma or other tumor types, BRAF-activating mutations are relatively rare events in HCC [9]. However, Raf kinase is overexpressed in a high percentage of human HCC tumors, and the RAF/MEK/ERK pathway can be activated by major etiologic factors such as hepatitis B virus (HBV) and hepatitis C virus (HCV) infection [9,10]. In a series of elegant experiments, Liu and colleagues demonstrated that sorafenib inhibited the phosphorylation of MEK and ERK and downregulated cyclin D1 levels in both PLC/PRF/5 and HepG2 cells [11]. Sorafenib also reduced the phosphorylation level of eIF4E and downregulated the antiapoptotic protein Mcl-1 in a MEK/ERK-independent manner. Consistent with the effects on both MEK/ERK-dependent and MEK/ERK-independent signaling pathways, sorafenib inhibited proliferation and induced apoptosis in both HCC cell lines. In the PLC/PRF/5 xenograft model, sorafenib at 30 mg/kg produced complete tumor growth inhibition. Mechanistically, sorafenib inhibited the phosphorylation of both ERK and eIF4E, reduced the microvessel area (assessed by CD34 immunohistochemistry), and induced tumor cell apoptosis (assessed by terminal deoxynucleotidyl transferase-mediated nick-end labeling) in PLC/PRF/5 tumor xenografts. These findings provide strong support that sorafenib could target HCC through inhibition of tumor angiogenesis and tumor cell proliferation.

The successful clinical development of sorafenib in HCC and the improved understanding of the mechanism of action of sorafenib have raised several critical questions. What are the potential mechanisms of action that lead to the sorafenib-mediated clinical benefits? Is this due to the VEGFR blockage, inhibition of RAF/MEK/ERK signaling pathway, or off target effects? What are the molecular and clinical predictors of benefits of sorafenib in HCC? In an article published this month in *BMC Medicine*, Zhang and colleagues provided evidence to support the notion that pERK might be a useful predictive marker in preclinical HCC model [3]. RAF/MEK/ERK is an important prototypical signal transduction pathway that is aberrantly activated in many malignancies [12]. The MEK homologues MEK1 and MEK2 are dual specificity kinases, uniquely sharing the consensus kinase motifs of both serine/threonine as well as tyrosine kinases. They exhibit a high degree of stringency in their ability to phosphorylate their ERK substrates. Both MEK isoforms sequentially phosphorylate ERK1 and ERK2 at two sites, initially Tyr^185 ^followed by Thr^183 ^[13]. Therefore, pERK is a key downstream component of the RAF/MEK/ERK pathway. Once phosphorylated, pERK can be translocated to the nucleus where it can regulate gene expression by phosphorylating and modulating various transcription factors and target genes [14]. The key strategic location of pERK provides the theoretical basis for its potential role to predict response to agents including sorafenib that inhibit the RAF/MEK/ERK pathway. Using different HCC cell lines (SMMC-7721, MHCC97-L, MHCC97-H and HCCLM6), Zhang *et al*. assessed the pERK expression by immunocytochemical quantification and found that basal pERK levels increased stepwise in these cell lines in accordance with their metastatic potential. Sorafenib inhibited ERK phosphorylation in a dose-dependent manner in these cell lines at a concentration between 5 and 20 μM and the degree of inhibition correlated with their basal pERK expression level. They further demonstrated that the effects of sorafenib on cell proliferation correlated significantly with basal pERK levels. To explore if this correlation was linked to pERK, the authors showed that by reducing the basal ERK phosphorylation level in MHCC97-H with U0126, a selective inhibitor of MEK1/2, they could render these cells less sensitive to sorafenib-mediated growth inhibition. These preclinical findings lend support to the initial report from the phase II study of sorafenib in advanced HCC, in which 33 out of 137 enrolled patients had pretreatment archived tumor tissue available for pERK staining and correlative analyses. In the majority of tumor samples, staining was generally most intense within the nucleus of tumor cells and there was a significant difference in TTP between patients with higher tumor cell pERK staining intensity, versus those with lower intensity [15].

Is the evidence strong enough to support the use of pERK as a predictive biomarker for sorafenib in HCC? Although this is an intuitive biomarker candidate for sorafenib, and the study of Zhang and colleagues provides promising *in vitro *results, much has to be done before pERK evaluation will become a useful biomarker. Future mechanistic study should assess the link of perturbing pERK pathway in each cell line with drug sensitivity. In addition, the expression of other signaling molecules should be evaluated to make certain that the sensitivity to sorafenib is indeed dependent on pERK expression. The clinical evidence to support the use of pERK as a predictive marker for sorafenib in HCC is also preliminary. Future studies should evaluate pERK expression in large numbers of patients and determine if this biomarker has predictive or prognostic value.

Identification of potential predictive and surrogate markers in patients receiving sorafenib and other targeted agents remains an area of active investigation. In a preliminary presentation at the American Association for the Study of Liver Diseases (ASSLD) meeting in 2008, Llovet and colleagues reported the early results of assessing potential useful predictive biomarkers in the SHARP study [16]. They found that sorafenib significantly decreased plasma levels of soluble c-KIT, sVEGFR2, sVEGFR3 and increased VEGF levels at 12 weeks. HCC patients with baseline high levels of soluble c-KIT showed a trend to better response to sorafenib in terms of OS and TTP. In addition, baseline higher pERK immunostaining correlated with a trend towards longer TTP. We are eagerly waiting the final report of the correlative analyses from this randomized study. Finally, in an attempt to evaluate the mechanisms of action and to identify useful biomarkers, extensive correlative studies have been performed in two phase II studies of sunitinib (a multitargeted tyrosine kinase inhibitor with overlapping but not identical spectrum with sorafenib) in HCC [17,18]. They identified associations between soluble c-KIT, interleukin (IL)6 and other cytokines and OS and progression free survival in advanced HCC patients. Collectively, these circulating biomarker data suggest a critical role for the balance between angiogenic and inflammatory pathways in HCC response and resistance to treatment with tyrosine kinase inhibitors. Successful modulation of these inflammatory markers might be critical for achieving treatment response with sunitinib and potentially other antiangiogenic agents including sorafenib.

In conclusion, the study by Zhang *et al*. provides additional evidence that baseline pERK may be a relevant marker to reflect the level of constitutive activation of the RAF/MEK/ERK signaling pathway and has the potential value in predicting response to sorafenib. The clinical data on the correlation of pERK and TTP is interesting, but preliminary. Whether baseline pERK will prove to be a useful predictive biomarker of response and clinical benefits for sorafenib in HCC will need to be validated in future large prospective studies.

## Competing interests

AZ works in an advisory role for Bayer and Genentech.

## Pre-publication history

The pre-publication history for this paper can be accessed here:


